# Willingness to vaccinate among adults, and factors associated with vaccine acceptance of COVID-19 vaccines in a nationwide study in Poland between March 2021 and April 2022

**DOI:** 10.3389/fpubh.2023.1235585

**Published:** 2023-12-04

**Authors:** Eftychia Kotronia, Magdalena Rosinska, Malgorzata Stepien, Michal Czerwinski, Malgorzata Sadkowska-Todys

**Affiliations:** ^1^Department of Epidemiology of Infectious Diseases and Surveillance, National Institute of Public Health - National Institute of Hygiene - National Research Institute, Warsaw, Poland; ^2^ECDC Fellowship Programme, Field Epidemiology Path (EPIET), European Centre for Disease Prevention and Control (ECDC), Stockholm, Sweden; ^3^Maria Sklodowska-Curie National Research Institute of Oncology, Warsaw, Poland

**Keywords:** vaccine hesitancy, SARS-CoV-2, Omicron variant, Delta variant, attitudes, COVID-19 waves, booster, vaccination campaign

## Abstract

**Introduction:**

Despite the availability, safety and effectiveness of COVID-19 vaccines, Poland remains one of the six countries of the European Union with the lowest cumulative uptake of the vaccine's primary course in the general population. This study examined willingness to vaccinate and the associated factors in samples of unvaccinated and vaccinated adults between March 2021 and April 2022.

**Methods:**

Data were collected using OBSER-CO, a nationwide, repeated cross-sectional study, conducted at four different time points (rounds). Data on willingness to vaccinate among the unvaccinated (at all rounds) and willingness to receive another dose in the vaccinated (at 2 rounds-after booster introduction), reasons for reluctance, sociodemographic, health, and behavioral factors were collected using a uniform questionnaire via computer-assisted telephone interviewing. In each round, more than 20,000 respondents were interviewed. To assess associations between factors and willingness to vaccinate, separate multivariable logistic regression models were fitted for each factor at each round and adjusted for confounders.

**Results:**

Between rounds 1 and 4 (March 2021–April 2022), in the unvaccinated, willingness to vaccinate declined from 73 to 12%, whereas in the vaccinated, willingness to receive another dose declined from 90 to 53%. The highest magnitude of decline between subsequent rounds occurred during the Omicron wave. Overall, concerns about side effects, effectiveness, and vaccine adverse effects were common but decreased over time. Age, gender, employment, place of residence, COVID-19 diagnosis or exposure, hospitalization, and participation in social activities were among the factors associated with willingness. However, associations changed over rounds highlighting the influence of different pandemic waves and variants.

**Conclusion:**

We observed a declining and multifactorial willingness to vaccinate in Poland, with vaccine attitudes dynamically changing across subsequent rounds. To address vaccine concerns, sustained health communication about COVID-19 vaccines is essential, especially after the emergence of new variants.

## Introduction

Early on during the pandemic, vaccines became one of the key preventive measures against COVID-19 (1, 2). In the last weeks of 2020, mass vaccination campaigns against COVID-19 were introduced in the European Union. In Poland, vaccination of individuals aged 60–64 years began in the last week of March 2021, and as of April 12, 2021, individuals younger than 60 could register to receive the vaccine. Additionally, on August 27, 2021, the Medical Council of Poland recommended administering the third (booster) dose of the vaccine to immunocompromised individuals. Vaccination with the booster dose began on September 1, 2021. As of September 23, 2021, booster vaccination was administered to individuals over 50 years of age, and as of November 2, 2021, to all adults. In the European Union (EU), Comirnaty, Valneva, Jcovden, Nuvaxovid, Spikevax, Vaxzevria, Bimervax, and VidPrevtyn Beta have been authorized for use by the European Medicines Agency (EMA). In Poland, most of the vaccinated population have received an mRNA vaccine.

Despite high availability of the vaccines through the National COVID-19 Immunization Program, as of 7th March 2023 in Poland, only 59.9% had completed the primary course (two doses), 33% had received the first, and 7.3% the second booster dose (3). The vaccine uptake in Poland is below the EU/EEA average in all vaccine categories (EU/EEA average for primary course: 73%; first booster: 54.7%; second booster: 14.1%). The difference in vaccination rates grows even bigger when compared with EU countries with the highest vaccination coverage, such us Portugal (86.4; 68.4; 30.3%, respectively) and Denmark (81.9; 62.8; 32.7% respectively) (3). Since February 2022, uptake of the primary course in Poland has remained stable, while uptakes of the first and second booster doses have been increasing at a very slow rate since their introduction (3).

Although vaccines have been proven to be effective against COVID-19 (4), willingness to vaccinate remains moderate worldwide and particularly in Poland (5, 6). Willingness to vaccinate is multifactorial and varies across countries (7). It can be influenced by demographic, psychological, and social/cultural factors (7–11). It has been shown consistently that being a woman, unemployment, and no prior COVID-19 infection are associated with higher reluctance to vaccinate (8, 9). Similarly, these factors affect uptake of booster doses. According to a recent meta-analysis of data from 23 different countries, age, gender, COVID-19 infection, work status, income, and health status were all predictors of willingness to receive a booster vaccine (12). Furthermore, concerns about the safety and effectiveness of vaccines have been prevalent in Poland since the beginning of the vaccination campaign (13). These concerns can impact an individual's decision to get vaccinated, and particularly it has been demonstrated that fear of side effects of COVID-19 vaccines and concerns about the speed of development or low trust in the effectiveness of the vaccine can negatively influence willingness to vaccinate in the adult population (7, 12, 14). In addition, since the start of the pandemic, the spread of misinformation and conspiracy theories about COVID-19 vaccines have hindered vaccine uptake and willingness to vaccinate (13, 15). As a result of the multifactorial nature of willingness to vaccinate and varying results across countries it is difficult to provide a single explanation behind the driving forces of willingness to vaccinate.

Importantly, these factors are unlikely to remain stable. Different COVID-19 variants and pandemic waves can heighten concerns and mistrust about vaccines (6). The severity of each variant (i.e., Delta, Omicron) can influence public opinion on the necessity of vaccination (16–18). While studies examining willingness to vaccinate/vaccine acceptance usually addressed the problem in one point in time, less is known about longitudinal changes in attitudes toward COVID-19 vaccines across different pandemic waves. To better understand the drivers behind willingness to vaccinate, it is important to disentangle how multifactorial associations evolved over the course of the COVID-19 pandemic. And more importantly, because of the unsatisfactory vaccination uptake in Poland, it is essential to gain insight into the reasons for reluctance to vaccinate over time. Findings can advise actions to boost vaccination but can also benefit health communication and vaccination campaigns, which can adjust their message according to the evolving concerns and specific characteristics of the population.

Therefore, this study aimed to examine willingness to vaccinate among unvaccinated individuals in four different time points (March 2021–April 2022; at least 2 months apart) and willingness to receive another dose of a COVID-19 vaccine among vaccinated individuals in two time points (November 2021–April 2022; after introduction of the booster vaccination campaign). Additionally, we aimed to investigate the reasons for reluctance to vaccinate in both vaccinated and unvaccinated participants, as well as the factors associated with willingness to vaccinate at 4 time points in unvaccinated participants in Poland. We focused on this period to examine the impact of different pandemic waves on willingness to vaccinate, especially Delta and Omicron waves. Due to the dynamic nature of this pandemic, beliefs and attitudes toward vaccinations were constantly shifting. Furthermore, we aimed to include key time points for COVID-19 vaccination, such as the introduction of COVID-19 primary and booster vaccination campaigns, and explore how their introduction influenced attitudes toward vaccination.

## Materials and methods

### Study design and participants

OBSER-CO is a nationwide, repeated cross-sectional study aiming to examine seroprevalence of COVID-19 antibodies, vaccination status and willingness to vaccinate in Poland. This study was based on the standardized protocol published by the World Health Organization (WHO) “*Population-based age stratified seroepidemiological investigation protocol for coronavirus 2019 (COVID-19) infection”* (WHO Unity studies) (19). This protocol provided guidelines for the investigation of the seroprevalence of COVID-19 antibodies and infection rates in the general population (19). However, each country could adjust the protocol according to specific country characteristics and additional research objectives. Details on study design, recruitment, and sampling can be found elsewhere (19, 20). Data collection took place at four different rounds, starting from March 2021 until April 2022. In particular, round 1 was carried out between 29th March and 14th May 2021, round 2 from 27th July to 7th September 2021, round 3 from 16th November to 23rd December 2021 and round 4 from 14th March 2022 to 26th April 2022. To monitor changes over time, the distance between former and next round was set to be at least 2 months. Sampling and recruitment of participants were performed by IPSOS. Participants were recruited randomly by Random Digit Dialing (RDD). Once the random sample was selected, it was stratified according to age and population distribution of each administrative region. For each region we aimed to recruit participants representative of the age distribution of the region. During random dialing if a prospective participant was part of an age group that we had already recruited the necessary number of participants, then this individual would not be invited to participate in the study.

In each round, data were collected through a telephone interview by trained interviewers (21). After the initial contact and once the individual had agreed to participate, a computer assisted telephone interview (CATI) was conducted. During the CATI, participants were asked about their willingness to vaccinate as well as demographics, household size, COVID-19 diagnosis, symptoms, sick leave, general and COVID-19 related hospitalization, exposure to COVID-19, and vaccination status. In comparison to the questionnaire supplied through the WHO protocol, we added items on demographic characteristics, vaccination status, willingness to vaccinate for both vaccinated and unvaccinated and reasons for reluctance to vaccinate in both subgroups. In our study, the questionnaire was developed by a research group based at the Department of Epidemiology and Surveillance of the National Institute of Public Health based on the questionnaire appended to the WHO Unity Protocol (20). Although the questionnaire was not validated, the items included were gathered from existing tools or have been already used in previous seroprevalence studies. Additionally, questions on reasons for reluctance to vaccinate were informed by published studies examining reasons for vaccine hesitancy of COVID-19 vaccines in Poland and worldwide. Furthermore, after the first two rounds we added questions about reinfections to account for repeated COVID-19 infections in individuals. Overall, after emergence of each variant questions were revised to ascertain that they reflected disease characteristics of each variant/pandemic wave. The detailed questionnaires used in round 1 and subsequent rounds can be found in Supplementary Text 1. At rounds 2, 3, and 4, alongside the recruitment of new individuals, participants from previous rounds were also invited to participate, resulting in a sample of new and panel participants. In round 1 data from 25,202 participants from the telephone interview were available; in round 2 from 21,503; in round 3 from 20,958; and in round 4 from 20,942 participants. The study was conducted according to the Declaration of Helsinki. Participants provided informed consent for their participation in the telephone interview. The study protocol was approved by the Bioethics Committee of the National Institute of Public Health NIH - National Research Institute (No. 5/2021 of 02/03/2021).

### Measures

#### Willingness to vaccinate among unvaccinated

To assess willingness to receive any COVID-19 vaccine at each round, participants were asked whether they were planning to get vaccinated. This question was asked only among unvaccinated individuals during the telephone interview. Available responses were yes or no. In rounds 2, 3, and 4 unvaccinated participants who responded no, were asked further about the reasons for their reluctance to vaccinate. They could choose one response from a set of reasons including: (1) I am concerned about the side effects/I am afraid of allergic reactions, (2) the vaccine was developed too quickly, it can't be safe, (3) the vaccine will be effective only for a short time and it will not protect against COVID-19 variants, (4) I do not vaccinate as a rule; I do not trust pharmaceutical companies (5) I got sick with COVID-19, (6) I faced difficulties enrolling at a vaccination center near my residence and I will not try again, (7) I faced difficulty reaching the vaccination center on my own; I am sick/unhealthy/unable to move, (8) I do not consider COVID-19 a dangerous disease, (9) I have a doctor's contraindication to vaccination, (10) I believe that getting sick is more effective than getting vaccinated, and (11) other reason. In round 4, participants could also opt out of responding to this question.

#### Willingness to receive another dose of a COVID-19 vaccine

Between rounds 2 and 3 the booster dose was recommended in the adult population. To estimate willingness to receive another dose of the COVID-19 vaccine, vaccinated participants were asked the following question: “*Are you planning to get vaccinated with another dose of the COVID-19 vaccine?”* in rounds 3 and 4. Participants could respond yes or no. Those responding no, were asked about the reasons for their reluctance to receive another dose. Participants were provided with the following reasons: (1) I got infected with COVID-19 despite being vaccinated, (2) I stopped believing in the effectiveness of the vaccine, (3) I felt bad after the previous vaccine dose (adverse effects), (4) The vaccine is only effective for a short period of time and it will not protect against variants, (5) I faced difficulties enrolling at a vaccination center near my residence, (6) I had difficulty reaching the vaccination center on my own; I am sick/unhealthy, (7) the doctor did not qualify me for vaccination due to health reasons, and (8) other reason. Participants could choose one reason.

### Factors

#### Sociodemographic

In all four rounds, sociodemographic factors included age, gender, work status, remote work, household size, and place of residence. Age consisted of four groups 20–39, 40–59, 60–69, and ≥70 years, and gender included man or woman. Work status comprised employed, and unemployed, whereas remote work was classified as remote or hybrid/stationary. For household size, participants were asked about the number of people included in their household, which ranged from 1 to ≥5 members. The participant's place of residence was based on population size and consisted of four levels: village, city up to 50,000 residents, city of 50,000–100,000 residents, and city of >100,000 residents. Additionally, in rounds 3 and 4, education was measured and consisted of low (primary, junior high school, basic vocational), medium and high (university degree, engineer degree, master's degree) level.

#### Infection with COVID-19

In all rounds, participants were asked whether they had received a positive COVID-19 test (PCR, antigen) since March 2020 (yes/no). In rounds 3, and 4 participants were additionally asked whether they had received more than one positive COVID-19 test results to account for new infections or re-infections (yes/no).

#### Exposure to COVID-19

Participants were asked whether they were in direct contact for at least 15 min with a person diagnosed with COVID-19 during the infectious period. Contacts with infectious individuals while wearing a mask, i.e., at least a FFP2 (N95) mask were not included in the contact group. Only contacts with infected individuals when wearing a cloth mask or only a face shield were included. Available responses consisted of yes, once; yes, multiple times; or no contact.

#### Symptoms

Participants were asked whether, in the previous 6 months, they had experienced any of the following symptoms: fever, cough, dyspnea, loss of smell or taste, sore throat, rhinorrhea, myalgia, fatigue, headache, abdominal pain, nausea or vomiting, diarrhea, rash, conjunctivitis, chills, loss of appetite, epistaxis (nosebleed), confusion, and other neurological symptoms. In rounds 3, and 4, other neurological symptoms were excluded, and instead participants were asked whether they experienced hearing problems. Participants could choose more than one symptoms. Then, a continuous variable for the number of symptoms was created, ranging from 0 to 18.

#### Sick leave, general hospitalization, hospitalization due to COVID-19

For sick leave, participants were asked whether they were on sick leave due to these symptoms (yes, no, or not applicable). For general hospitalization, participants were asked whether they had been hospitalized since March 2020 (yes/no). If participants were hospitalized for any reason, then they would be further asked about COVID-19 related hospitalization. To assess hospitalization due to COVID-19, participants were asked whether they were hospitalized due to COVID-19 or a respiratory infection (pneumonia, bronchitis). Available responses were yes, no, or not applicable. This question was not asked to participants who did not report general hospitalization.

#### Participation in activities

Participants were asked three separate questions about participation in specific social activities. Individuals were asked whether, since March 2020 (rounds 1 and 2) or May 2021 (rounds 3, and 4), they took part in events such as weddings, communions, baptisms, and/or funerals (yes/no). Similarly, participants were asked whether they regularly participated in sports, religious, artistic groups, or similar activities/meetings, not related to work (yes/no). Finally, individuals were asked if they participated in organized trips (i.e., trip or camping, business trip, sports trip) (yes/no).

### Statistical analysis

Prevalence of willingness to vaccinate was defined as the percentage of participants, who responded that they were planning to get vaccinated or receive another dose of COVID-19 vaccine at each round. Demographic variables as well as reasons for reluctance to vaccinate were summarized as proportions with percentages. Aside from symptoms which was coded as a continuous variable (number of symptoms), all other factors were categorical (binary, nominal, ordinal). To analyze the factors associated with willingness to vaccinate in unvaccinated participants we performed the following steps. Factors of interest were chosen according to previous research examining variables associated with COVID-19 disease characteristics, vaccination and willingness to vaccinate. These factors included age, gender, place of residence, work status, remote work, COVID-19 diagnosis, exposure to COVID-19, general hospitalization, hospitalization due to COVID-19, participation in events, participation in social groups, and participation in organized trips. Secondly, we performed univariate analysis for the selected factors in each round separately. Factors which were not associated with willingness to vaccinate in univariate analysis were not examined further. Finally, multiple multivariable logistic regression models were created to examine these associations. At each round, for each of the selected factors a separate regression model was fitted, which was adjusted for a different set of confounders. A detailed list of confounders for each regression model can be found in Supplementary Table 1. Each set of confounders was selected according to previous literature about COVID-19 in general and COVID-19 vaccination behaviors. We also used the maximum likelihood estimate of the model to check how well each confounder fitted the regression model. We selected this approach instead of a single multivariable model to build the most appropriate model for each exposure, accounting for the fact that each exposure can be influenced by different confounders (22). Additionally, we performed separate analyses for each round, to observe the effects of different COVID-19 variants and subsequent pandemic waves.

Effect estimates are presented as odds ratios (OR), crude for univariate and adjusted for the multivariable analysis, with corresponding 95% confidence intervals (CI). Regression analyses were bootstrapped (1,000 repetitions) and the Bonferroni correction was applied to correct confidence intervals for multiple comparisons. Analyses were performed using STATA 14 (College Station, TX: StataCorp LP).

## Results

In total, 92,607 CATI interviews were conducted. In round 1 (R1), 63% were unvaccinated (*n* = 15,885), and 27% were vaccinated (*n* = 9,317). In round 2 (R2), 18.6% were unvaccinated (*n* = 4,006), whereas 81.4% were vaccinated (*n* = 17,497). In round 3 (R3), 14.5% were unvaccinated (*n* = 3,044) and 85.5% vaccinated (*n* = 17,914). Finally, in round 4 (R4), 19.3% were unvaccinated (*n* = 4,032) and 80.7% were vaccinated (*n* = 20,942).

### Characteristics of unvaccinated participants according to willingness to vaccinate

By round, among unvaccinated participants, there were respectively 7,991 (59%), 1,934 (17%), 1,388 (12.9%), 1,975 (15.4%) women with median age 47 years (40–62) in R1, 47 (36–61) in R2, 44 (34–62) in R3, and 45 (35–60) in R4. Characteristics of unvaccinated study participants according to their willingness to vaccinate at each round are presented in Table 1. Among unvaccinated individuals, willingness to vaccinate was 73% in R1. In R2, 3 months after vaccination became available to all adults, willingness fell to 37%. In R3, willingness decreased slightly to 32%, and in R4, after the emergence of Omicron, willingness to vaccinate fell to 12%.

**Table 1:** Characteristics of unvaccinated participants according to willingness to vaccinate in rounds 1–4.

	**Willingness to vaccinate**															
	**Round 1 (*****n*** = **15,885)**				**Round 2 (*****n*** = **4,006)**				**Round 3 (*****n*** = **3,044)**				**Round 4 (*****n*** = **4,023)**			
	**Yes (*****n*** = **11,596, 73%)**		**No (*****n*** = **4,289, 27%)**		**Yes (*****n*** = **1,482, 37%)**		**No (*****n*** = **2,524, 63%)**		**Yes (*****n*** = **974, 32%)**		**No (*****n*** = **2,070, 68%)**		**Yes (*****n*** = **483, 12%)**		**No (*****n*** = **3,540, 88%)**	
	***N***	**(%)**	***N***	**(%)**	***N***	**(%)**	***N***	**(%)**	***N***	**(%)**	***N***	**(%)**	***N***	**(%)**	***N***	**(%)**
**Age in years**																
20–39	3,759	(65)	1,996	(35)	614	(34)	1,196	(66)	439	(29)	1,060	(71)	217	(12)	1,567	(88)
40–59	4,857	(76)	1,524	(24)	504	(38)	817	(62)	293	(34)	571	(66)	184	(13)	1,250	(87)
60–69	2,135	(79)	563	(21)	236	(42)	329	(58)	178	(41)	258	(59)	67	(12)	483	(88)
≥70	810	(77)	241	(23)	131	(42)	179	(58)	80	(33)	165	(67)	35	(13)	229	(87)
**Gender**																
Man	5,770	(73)	2,124	(27)	736	(36)	1,336	(64)	484	(29)	1,172	(71)	229	(11)	1,828	(89)
Woman	5,791	(72)	2,200	(28)	749	(39)	1,185	(61)	506	(36)	882	(64)	274	(14)	1,701	(86)
**Place of residence**																
Village	3,312	(70)	1,422	(30)	588	(42)	816	(58)	373	(36)	654	(64)	182	(14)	1,164	(87)
City up to 50,000 inhabitants	2,810	(72)	1,120	(28)	394	(37)	661	(63)	227	(29)	544	(71)	132	(13)	898	(87)
City 50,000–100,000 inhabitants	897	(72)	351	(28)	117	(34)	224	(66)	84	(29)	203	(71)	36	(11)	293	(89)
City >100,000 inhabitants	4,542	(76)	1,431	(24)	386	(32)	820	(68)	306	(32)	653	(68)	153	(12)	1,174	(88)
**Size of household**																
1	1,911	(74)	661	(26)	283	(40)	429	(60)	213	(36)	380	(64)	118	(15)	673	(85)
2	3,401	(76)	1,054	(24)	361	(35)	676	(65)	224	(31)	499	(69)	110	(12)	799	(88)
3	2,432	(74)	871	(26)	323	(39)	510	(61)	200	(32)	422	(68)	107	(13)	695	(87)
4	2,357	(71)	967	(29)	281	(35)	512	(65)	188	(31)	420	(69)	87	(11)	727	(89)
5 or more	1,460	(65)	771	(35)	237	(38)	394	(62)	165	(33)	333	(67)	81	(11)	635	(89)
**Work status**																
Employed	6,281	(72)	2,448	(28)	733	(35)	1,368	(65)	469	(30)	1,105	(70)	236	(11)	1,913	(89)
Unemployed	1,047	(65)	555	(35)	211	(41)	308	(59)	144	(37)	244	(63)	96	(18)	435	(82)
**Pensioner**																
Yes	2,756	(79)	753	(21)	366	(42)	502	(58)	248	(38)	399	(62)	111	(14)	697	(86)
No	8,805	(71)	3,571	(29)	1,119	(36)	2,019	(64)	742	(31)	1,655	(69)	392	(12)	2,832	(88)
**COVID-19 diagnosis**																
Yes	2,091	(79)	554	(21)	253	(49)	267	(51)	768	(31)	1,710	(69)	39	(13)	264	(87)
No	9,470	(72)	3,770	(29)	1,232	(35)	2,254	(65)	222	(39)	344	(61)	464	(12)	3,265	(88)
**Contact with infected individual**																
Once	1,363	(75)	446	(25)	131	(43)	173	(57)	86	(32)	187	(68)	63	(13)	414	(87)
Multiple times	2,047	(72)	784	(28)	188	(28)	484	(72)	101	(27)	273	(73)	76	(8)	888	(92)
No contact	8,151	(72)	3,094	(28)	1,166	(38)	1,864	(62)	803	(34)	1,594	(66)	364	(14)	2,227	(86)
**General hospitalization**																
Yes	1,118	(78)	317	(22)	171	(41)	246	(59)	144	(40)	218	(60)	87	(17)	411	(83)
No	10,443	(72)	4,007	(28)	1,314	(37)	2,275	(63)	846	(32)	1,836	(68)	416	(12)	3,118	(88)
**Hospitalization due to COVID-19**																
Yes	180	(88)	25	(12)	29	(56)	23	(44)	17	(50)	17	(50)	25	(30)	58	(70)
No	11,381	(73)	4,299	(27)	1,456	(37)	2,498	(63)	973	(32)	2,037	(68)	478	(12)	3,471	(88)
**Participation in events**																
Yes	3,637	(69)	1,597	(31)	593	(32)	1,278	(68)	413	(28)	1,054	(72)	155	(9)	1,597	(91)
No	7,924	(74)	2,727	(26)	892	(42)	1,243	(58)	577	(37)	1,000	(63)	348	(15)	1,932	(85)
**Participation in social groups**																
Yes	2,288	(65)	1,249	(35)	383	(29)	955	(71)	285	(26)	819	(74)	154	(9)	1,507	(91)
No	9,273	(75)	3,075	(25)	1,102	(41)	1,566	(59)	705	(36)	1,235	(64)	349	(15)	2,022	(85)
**Participation in organized trip**																
Yes	1,597	(68)	744	(32)	222	(27)	600	(73)	179	(25)	540	(75)	70	(8)	821	(92)
No	9,964	(74)	3,850	(26)	1,263	(40)	1,921	(60)	811	(35)	1,514	(65)	433	(14)	2,708	(86)

Across all sociodemographic and other groups studied, willingness to vaccinate followed a declining pattern from R1 to R4. In R1, R2, and R3 middle-aged and older individuals were more likely to express willingness to vaccinate than younger participants. However, in R4, there were no differences among age groups. In the first 2 rounds, willingness to vaccinate was similar between men and women. However, in R3 (Delta wave/booster vaccination), and R4 (Omicron) women reported willingness to vaccinate more often than men. No clear pattern was observed for place of residence or size of household. For work status, in R1 72% of employed participants reported willingness to vaccinate compared to 65% of unemployed. Nevertheless, from round 2 onwards, unvaccinated, unemployed individuals were more likely to be willing to get vaccinated compared to employed. Additionally, a clear pattern was present for pensioners in all four rounds. Pensioners were consistently more willing to get vaccinated compared to non-pensioners. However, the size of the difference decreased from R3 to R4, after the emergence of the Omicron variant. A similar pattern was observed for COVID-19 diagnosis. Those with a positive COVID-19 test were more likely to be willing to vaccinate in the first three rounds (R1: 79%; R2: 49%; R3:39%) compared to those without a COVID-19 diagnosis (R1: 72%; R2: 35%; R3: 31%). But in R4, no difference between group levels was present. In R2, R3, and R4 those who had multiple contacts with an infected person reported a lower willingness to vaccinate compared to those who had no or just one contact. Overall, in all rounds prevalence of willingness to vaccinate was higher in those hospitalized (general or COVID-19 related). Finally, in all four rounds, individuals who did not participate in events, social groups, and organized trips, were more likely to express willingness to vaccinate than those who participated.

### Reasons for reluctance to vaccinate among unvaccinated participants

**Figure 1:**
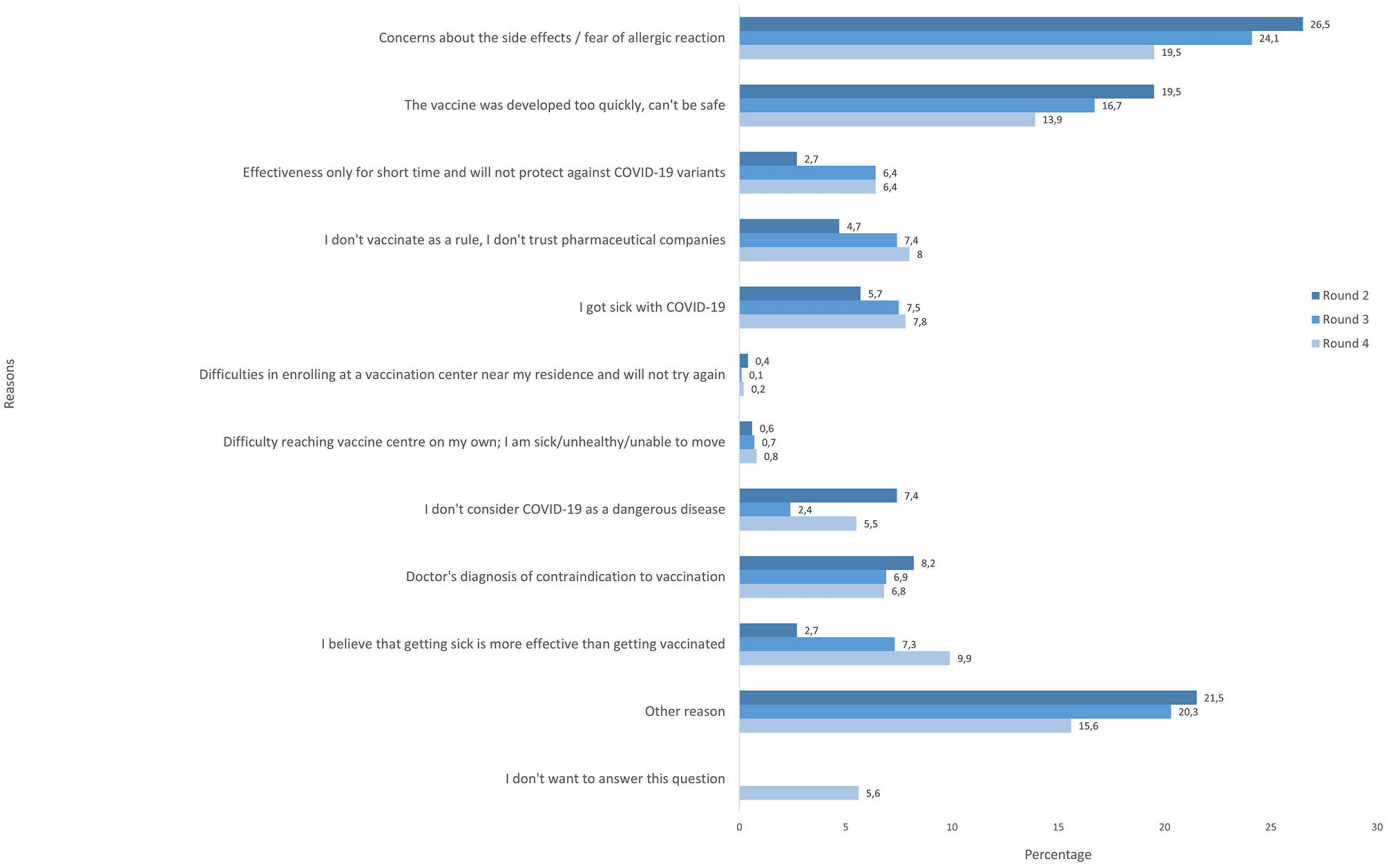
Reasons for reluctance to vaccinate among unvaccinated participants in rounds 2–4.

Figure 1 presents the reasons for reluctance to vaccinate among unvaccinated participants at R2, R3, and R4. In R2, 26.5% of participants expressed concern about the side effects of the COVID-19 vaccine as the reason for reluctance to vaccinate. In R3, concerns about the side effects and allergic reactions declined but remained the most common reason with 24.1%. Similarly, in R4, the percentage of participants expressing concerns about side effects or allergic reactions continued to decline but these concerns remained one of the most prevalent. Furthermore, in R2, 19.5% mentioned that the vaccine was developed too quickly and expressed concerns about its safety. In R3, 16.7% reported this reason, which further declined in R4 to 13.9%. Additionally, in R2, 8.2% of participants reported a doctor's diagnosis of contraindication, 7.4% said that they did not consider COVID-19 a dangerous disease, 5.7% cited COVID-19 infection, 4.7% responded that they do not trust pharmaceutical companies in general, and 2.7% thought that getting infected is more effective than vaccination. In R3, more people chose COVID-19 infection (7.5; 1.8% increase since R2), 7.3% responded that getting sick is more effective than being vaccinated (4.6% increase since R2), 6.9% chose medical contraindications, and 7.4% lacked trust in pharmaceutical companies and vaccines in general (2.7% increase since R2). In R4, 9.9% of respondents reported that getting sick is more effective than the vaccine, a reason that has become more prevalent since R3. Additionally, 7.8% of participants mentioned getting sick with COVID-19, 8% of people mentioned lack of trust against pharmaceuticals (further increase since R3), and 5.5% did not consider COVID-19 dangerous. In R2, R3, and R4 a significant proportion (R2: 21.5%; R3: 20.3%; R4: 15.6%) of participants chose “other reason”. In R4, 6% of participants did not want to answer this question.

### Characteristics of vaccinated participants according to willingness to receive another dose of a COVID-19 vaccine

There were 17,914 vaccinated individuals in R3 and 20,942 in R4, including 9,347 (52.2%) and 10,868 (51.9%) women with median age 62 years (43–70) and 57 (41–68) in R3 and R4, respectively. Table 2 presents the characteristics of respondents according to willingness to receive another dose in R3 and R4. A decline in willingness to receive another dose of a vaccine was observed from R3 to R4. In R3, 90% of vaccinated participants reported that they were willing to receive another dose. Among those, 91% had so far received 2 doses and 9% one dose of a COVID-19 vaccine. In R4, willingness decreased to 53%. Among those, 86% had so far received two doses and 14% one dose of a COVID-19 vaccine. Across all factors, willingness among the vaccinated declined substantially from R3 to R4. For age, willingness to receive another dose was the lowest among 20–39 years (R3: 85%; R4: 51%). Those ≥60 years reported the highest willingness to receive another dose in both rounds. Women consistently expressed higher willingness to receive another dose than men, while there was no clear pattern for place of residence. For work status, there was no big difference between employed and unemployed in R3 (88 and 89%, respectively), however this changed in R4. While willingness to vaccinate declined in both groups, it remained higher among the unemployed. Furthermore, vaccinated pensioners were more likely to express willingness than non-pensioners in both rounds. Furthermore, in R3, no differences were present in willingness according to COVID-19 diagnosis. However, in R4, those with a positive COVID-19 test reported higher willingness to receive another dose (60%) when compared to 52% of those without a positive test.

**Table 2:** Characteristics of vaccinated participants according to willingness to receive another dose of a COVID-19 vaccine in rounds 3-4.

	**Willingness to receive another dose of a COVID-19 vaccine**							
	**Round 3 (*****n*** = **12,573)**				**Round 4 (*****n*** = **7,105)**			
	**Yes (*****n*** = **11,347, 90%)**		**No (*****n*** = **1,226, 10%)**		**Yes (*****n*** = **3,775, 53%)**		**No (*****n*** = **3,330**, ***n*** = **47%)**	
	***N***	**(%)**	***N***	**(%)**	***N***	**(%)**	***N***	**(%)**
**Age in years**								
20–39	3,557	(85)	643	(15)	1,505	(51)	1,463	(49)
40–59	3,254	(91)	331	(9)	1,338	(54)	1,156	(46)
60–69	3,108	(96)	136	(4)	581	(56)	458	(44)
≥70	1,428	(92)	116	(8)	351	(58)	351	(42)
**Gender**								
Man	5,439	(89)	679	(11)	1,857	(51)	1,753	(49)
Woman	5,908	(92)	547	(8)	1,918	(55)	1,577	(45)
**Place of residence**								
Village	3,177	(90)	354	(10)	1,047	(52)	970	(48)
City up to 50,000 inhabitants	2,946	(91)	301	(9)	907	(52)	580	(48)
City 50,000–100,000 inhabitants	959	(92)	87	(8)	325	(56)	259	(44)
City >100,000 inhabitants	4,265	(90)	484	(10)	1,496	(54)	1,251	(46)
**Size of household**								
1	2,118	(91)	221	(9)	697	(52)	639	(48)
2	3,842	(93)	307	(7)	1,049	(56)	836	(44)
3	2,230	(89)	273	(11)	771	(54)	654	(46)
4	1,911	(87)	289	(13)	755	(51)	724	(49)
5 or more	1,246	(90)	136	(10)	503	(51)	477	(49)
**Work status**								
Employed	5,372	(88)	736	(12)	2,097	(55)	1,993	(60)
Unemployed	712	(89)	87	(11)	364	(10)	250	(7)
**Pensioner**								
Yes	4,120	(95)	230	(5)	857	(57)	655	(43)
No	7,227	(88)	996	(12)	2,918	(52)	2,675	(48)
**Positive COVID-19 test**								
Yes	2,084	(91)	197	(9)	391	(60)	265	(40)
No	9,263	(90)	1,029	(10)	3,384	(52)	3,065	(48)
**Participation in organized trip**								
Yes	3,195	(28)	408	(33)	844	(22)	861	(26)
No	8,152	(71)	818	(67)	2,931	(78)	2,469	(74)
**Number of vaccine doses received**								
1 dose	1,041	(78)	318	(23)	532	(42)	724	(22)
2 doses	10,306	(92)	908	(8)	3,243	(55)	2,606	(45)

### Reasons for reluctance to receive another dose of a COVID-19 vaccine among vaccinated participants

**Figure 2:**
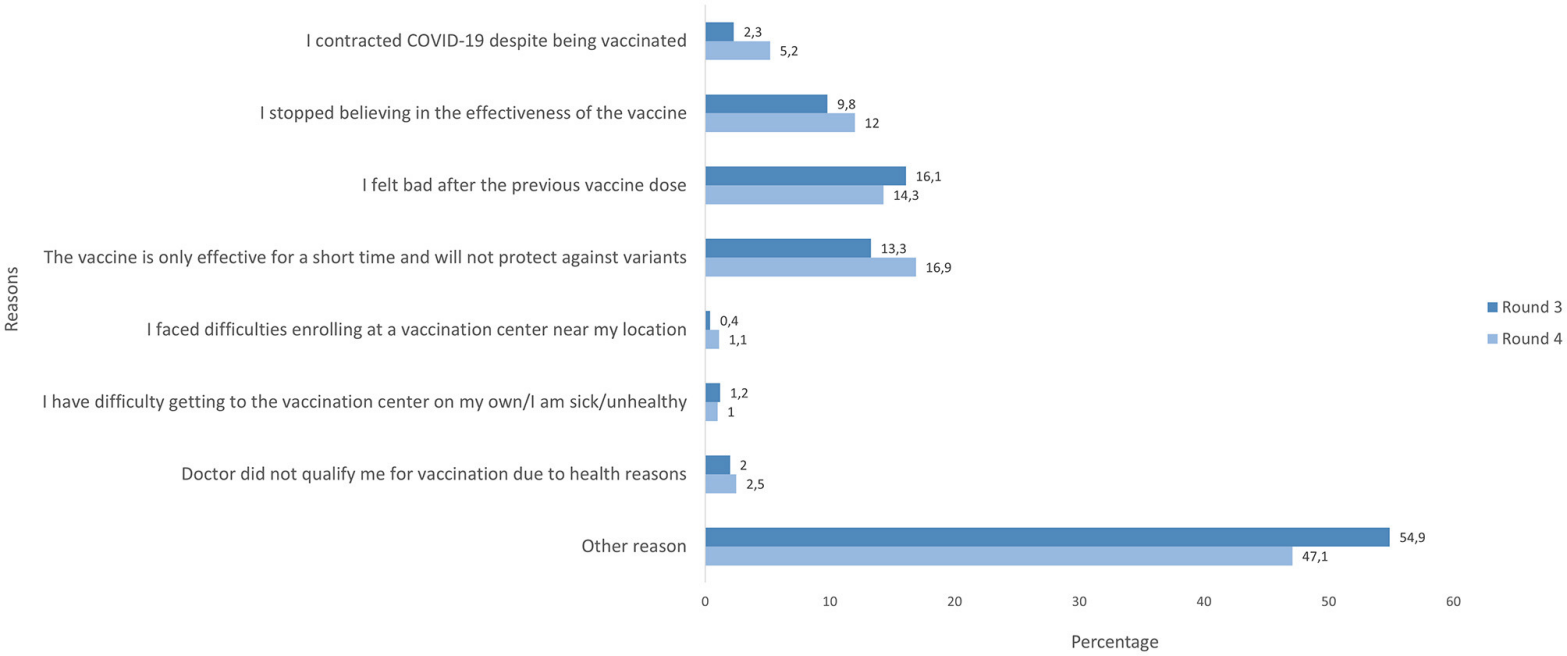
Reasons for reluctance to receive another dose of a COVID-19 vaccine among vaccinated participants in rounds 3–4.

Reasons for reluctance to receive another dose of a COVID-19 vaccine are presented in Figure 2. In R3, 16% of participants chose vaccine adverse effects (feeling bad after the previous vaccine dose). In R4, the prevalence of vaccine adverse effects declined to 14%. Additionally, in R2, 13% believed that the vaccine is only effective for a short period of time and will not protect against variants, while 10% stopped believing in the effectiveness of the vaccine altogether. In R4, more participants believed that the vaccine would only effective for a short period of time (17%), while 12% reported that they did not believe in the vaccine's overall effectiveness. In R3, only 2% of participants mentioned that they were reluctant to receive another dose because they were infected with COVID-19 after vaccination. This reason became more prevalent in R4 and increased to 5%. In R3, 55% of respondents chose “other reason” whereas in R4, there was a small decline with 47% citing “other reason”.

### Factors associated with willingness to vaccinate among unvaccinated participants

**Figure 3:**
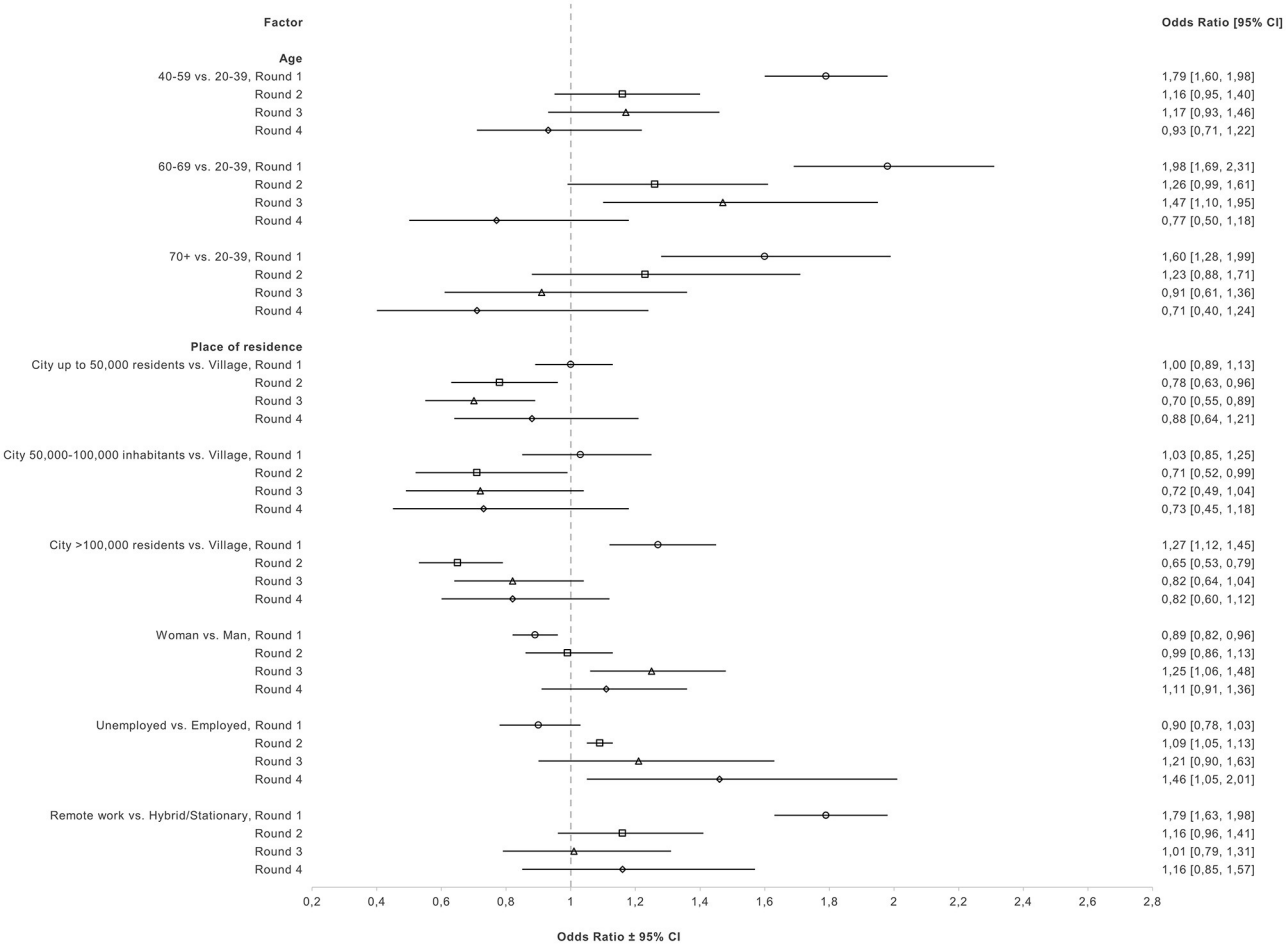
Fully adjusted odds ratios and 95% CI for associations of sociodemographic factors with willingness to vaccinate among unvaccinated participants in rounds 1–4.

Fully adjusted odds ratios (OR) and 95% CIs of associations of age, place of residence, gender, work status, and remote work with willingness to vaccinate among unvaccinated participants are presented in Figure 3. Detailed crude and fully adjusted ORs and 95% CIs can be found in Supplementary Table 2. Estimates for the association between age and willingness to vaccinate varied over time (rounds) and age groups. In R1, 40–59 years vs. 20–39 (reference), was associated with increased odds of willingness to vaccinate (OR = 1.79, 95% CI: 1.62–1.99) in the fully adjusted model. This association was attenuated in R2, R3, and R4. Furthermore, 60–69 years was associated with increased odds of willingness at R1 (start of primary vaccination) and R3 (booster vaccination) (OR = 1.98, 95% CI: 1.69–2.31; OR = 1.47, 95% CI: 1.10–1.95, respectively). At R2, and R4 no associations were observed between this age group and willingness to vaccinate. Finally, for those ≥70 years, a positive association was reported only in R1 (OR = 1.60, 95% CI: 1.28–1.99), with the association attenuating over the next 3 rounds.

In R1, living in a city of >100,000 residents (vs. a village) was associated with a higher willingness to vaccinate (OR = 1.27, 95% CI: 1.12–1.45); however this association reversed in the next three rounds with those living in big cities having decreased odds of willingness (R2, OR = 0.65, 95% CI: 0.53–0.79; R3, OR = 0.82, 95% CI: 0.64–1.04; R4: OR = 0.82, 95% CI: 0.60–1.12). Similar associations were observed from R2–R4 in those living in (a) cities up to 50,000 residents and (b) cities from 50,000 to 100,000 residents when compared to living in a village, after adjustment for confounders.

In R1, being a woman was associated with decreased odds of willingness (OR = 0.89, 95% CI: 0.82–0.96) when compared to men, after adjustment. In R2 we did not observe any association between gender and willingness. However, in R3, women had higher odds of willingness to vaccinate than men (OR = 1.25, 95% CI: 1.06, 1.48). But, in R4, this association was attenuated (OR = 1.11, 95% CI: 0.91–1.36). For work status, a negative association was observed for unemployed participants (vs. employed) in R1 (OR = 0.90, 95% CI: 0.78– 1.03). However, this association was reversed in the following 3 rounds, with unemployed participants reporting increased odds of willingness to vaccinate. Moreover, remote work was associated with increased odds of willingness to vaccinate when compared to hybrid/stationary work in R1 (OR = 1.79, 95% CI: 1.63–1.98). However, the association was attenuated in the next 3 rounds.

**Figure 4:**
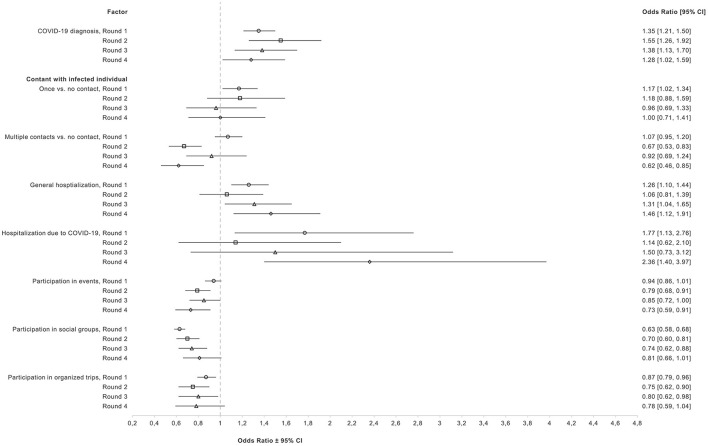
Fully adjusted odds ratios and 95% CI for associations of health, and behavioral factors with willingness to vaccinate among unvaccinated participants in rounds 1–4.

Fully adjusted ORs and 95% CIs of associations of COVID-19 diagnosis, exposure to COVID-19, general hospitalization, hospitalization due to COVID-19, participation in events, social groups, and organized trips with willingness to vaccinate are presented in Figure 4. Detailed crude and fully adjusted ORs and 95% CIs can be found in Supplementary Table 2. COVID-19 diagnosis was consistently associated with higher odds of willingness to vaccinate when compared to no diagnosis in all four rounds after adjustment for confounders (R1, OR = 1.35, 95% CI: 1.21–1.50; R2, OR = 1.55, 95% CI: 1.26–1.92; R3, OR = 1.38, 95% CI: 1.13–1.70; R4, OR = 1.28, 95% CI: 1.02–1.59). Moreover, having had contact once with an infected individual was positively associated with willingness to vaccinate in R1 (OR = 1.17, 95% CI: 1.02–1.34) in the fully adjusted model. A similar association was observed in R2 but was further attenuated in subsequent rounds. For multiple contacts with infected individual(s), associations changed from R1 (OR = 1.07, 95% CI: 0.95–1.20) to R2 (OR = 0.67, 95% CI: 0.53–0.83). In R3 the association was attenuated, but became stronger in R4 (OR = 0.62, 95% CI: 0.46–0.85). Moreover, general hospitalization and hospitalization due to COVID-19 were associated with increased odds of willingness to vaccinate in R1 (OR = 1.26, 95% CI: 1.10–1.44; OR = 1.77, 95% CI: 1.13–2.76, respectively). However, associations were attenuated in R2. In R3 the odds ratio for general hospitalization was equal to 1.31 (95% CI: 1.04–1.65) and for COVID-19-related hospitalization to 1.50 (0.73–3.12). In R4 those hospitalized for any reason had an OR of 1.46 (95% CI: 1.12–1.91) and those hospitalized because of COVID-19 had an OR of 2.36 (95% CI: 1.40–3.97). Lastly, participation in events, social groups, and organized trips were consistently associated with decreased odds of willingness to vaccinate in all 4 rounds after adjustment for confounders.

## Discussion

Our study documents the change in attitudes toward getting vaccinated or receiving an additional dose of a COVID-19 vaccine from March 2021 to April 2022 in Poland. Willingness to vaccinate among unvaccinated participants exceeded 70% in the first round but declined substantially in the next 3 rounds. Among those who remained unvaccinated until April 2022, only 12% planned to vaccinate in the future. This may be explained on one hand by the fact that the individuals, who planned to vaccinate already had a chance to do so, and on the other hand by decreasing overall interest to get vaccinated. The latter is also supported by the fact that, among vaccinated individuals, willingness to receive an additional dose of a COVID-19 vaccine also declined, although not to such a large extent. In round 3, November-December 2021, 90% of vaccinated participants intended to receive another dose, but after 4 months, in round 4, this percentage decreased to 53%. The decline in willingness to vaccinate over the study period among both vaccinated and unvaccinated individuals was observed across all sociodemographic, health, and behavioral factors examined.

The initial percentage of unvaccinated participants who intended to vaccinate against COVID-19 in our study (73%) was higher than reported by other authors. A study examining data from 2020 showed that Poland had one of the lowest vaccine acceptance rates (56.3%) (5), while other Polish studies reported vaccine hesitancy or reluctance to vaccinate varying between 31 and 49.2% in 2021 (7, 23). Although our study followed a random digit dialing recruitment the respondents who agreed to participate were clearly more inclined to vaccinate as the proportion of vaccinated in our study exceeded the population statistics. For example, in rounds 2–4 over 80% of participants were vaccinated with at least one dose, but in the official statistics this percentage reached only slightly above 60%, which is why we focused on separate analysis of vaccinated and unvaccinated cohorts. On the other hand differences in data collection (time, sample size, population characteristics) can potentially explain differences as compared with other research studies. A previous study, conducted at the start of the vaccination program reported increasing trend in willingness to vaccinate (14) so it is possible that our first round occurred at the time of the highest acceptance of the COVID-19 vaccines, which declined afterwards.

Of note, studies in the US conducted between 2020–2021, and 2021–2022 indicate that it is possible to maintain an increasing trend in willingness to vaccinate, although in contrast to our study this analysis included both the vaccinated and planning to get vaccinated as willing to vaccinate (24, 25). Their findings also indicate the positive impact of a number of interventions such as releasing restrictions and mask mandates for vaccinated individuals (24). Poland did not fully implement such approach and possibly in effect the pressure to vaccinate was less (26).

What is more, with longer duration of the COVID-19 emergency situation, the intention to vaccinate may be undermined by pandemic fatigue (6, 27). This could partially explain the decreasing trend in willingness to vaccinate observed in our study along with evolution of specific concerns regarding the vaccines and the infection itself. We note that the concerns were fueled by the increase of misinformation around the safety and efficacy of COVID-19 vaccines in the EU, giving rise to conspiracy theories (11), which then negatively influenced perceptions of vaccines. It is highly possible that the decreasing levels of willingness to vaccinate over time in our study also reflected the impact of the COVID-19 infodemic, especially driven by social media in Poland (7).

In rounds 2–4, we were able to collect data on reasons why the participants were reluctant to vaccinate confirming common themes of social media discourse (13). In accordance with previous studies in Poland, concerns about side effects/allergic reactions (28) and speed of development/safety were the most prevalent reasons for reluctance to vaccinate (14), listed by 26.5 and 19.5% of vaccine hesitant participants in round 2, respectively. However, there was a decreasing trend in these concerns over the study period. As time passed and more data became available about the safety of COVID-19 vaccines and in conjunction with communication efforts by public health authorities, concerns subsided, but nevertheless remained significant. In round 4 still 19.5% of participants stated concerns about side effects and 13.9% stated quick vaccine development/safety as key reasons for reluctance to vaccinate.

Furthermore, the belief that the effectiveness of the vaccine is limited and will not protect against new variants became more popular. The proportion of unvaccinated reluctant to vaccinate due to this reason changed from 2.7 to 6.6% between round 2 and 4 and the proportion of vaccinated not willing to take additional dose—from 13.3 to 16.9%. Interestingly, the rate of decrease of willingness to vaccinate was the largest after the Omicron wave across all factors. Moreover, in unvaccinated participants, after the emergence of the Omicron variant (R4) differences among levels of several factors disappeared, which could suggest strong influence of the Omicron wave on attitudes toward COVID-19 vaccination. The Omicron epidemic wave was characterized by very high transmission rates in combination with lowered vaccine effectiveness against mild infection (29–31). This could have contributed to increasing beliefs of lack of effectiveness or only short-lived effectiveness of the COVID-19 vaccine observed in our study, despite the fact of clear evidence of high vaccine efficacy against severe disease.

Additionally, the belief that the Omicron variant was not as severe as previous variants could also explain the increasing proportion of participants in our study believing that getting sick is more effective than getting vaccinated. Concerns about vaccine effectiveness were also identified as crucial for vaccine acceptance in other studies (10, 23, 32). Similarly, vaccinated participants in our study cited frequently vaccine-related adverse effects (16.1% in R3 and 14.3% in R4) and mistrust about the effectiveness of the vaccine in general (9.8% in R3 and 12.0% in R4) as reasons behind reluctance to receive another dose. Reduced effectiveness of the first mRNA vaccines against the Omicron variant and the increased number of Omicron infections in vaccinated people may be the driving forces behind these responses (10, 33). These findings highlight the importance of continuing health communication adjusted to the current concerns and incorporating new scientific developments (6). Of note, a substantial proportion of participants chose “other reason” as their response (15.6–21.5% among unvaccinated and 47.1–54.9% among vaccinated). The list of reasons provided to participants in our study, were chosen according to previous literature. The fact that so many participants did not find it sufficient underscores the dynamic nature of this pandemic and beliefs and attitudes toward vaccination, and the necessity to continuously evaluate new reasons behind reluctance in order to update the communication strategies.

Equally important, our study helps to better characterize the changing population who is reluctant to vaccinate or to receive another dose. In our study associations between age and willingness to vaccinate varied between rounds. Initially, there was a strong association with age group, with older unvaccinated individuals more likely to be willing to receive the vaccine in the future. This is in line with prior research indicating that older individuals are more likely to get vaccinated (7) and less likely to delay getting the vaccine compared to younger individuals (25). Middle-aged and older individuals tend to have higher risk perception toward COVID-19 and higher engagement with preventive measures (34), which explains the initial finding. However, another study in Poland did not report any associations between age and willingness to vaccinate (35). We observed that the difference between age groups decreased in subsequent rounds, so evolution in time of the reluctant group may explain contrasting findings reported in previous literature.

Moreover, in round 1, women had decreased odds of willingness to vaccinate, in accordance with another study in Poland (36). Women in general experience more vaccine-related adverse effects than men, which can explain the increased reluctance and could potentially reflect increased fear toward COVID-19 vaccination (37). However, by round 3 (November-December 2021; Delta/Omicron) women were more willing to get vaccinated than men. Potentially fears of women subsided, as vaccines proved to be safe and effective, but it is also likely that women who intend to vaccinate in general, were delaying getting the vaccine, while those men who wanted to get vaccinated, did so. Similar mechanism could explain the changes of the association between place of residence and willingness to vaccinate throughout the study period. In round 1, participants living in big cities were more willing to get vaccinated than those living in villages, a finding which is in accordance with previous studies in Poland (14, 35, 36). However, we observed a reversal in associations of all levels of place of residence compared to living in a village in rounds 2–4. During all four rounds, we observed the lowest rates of vaccine uptake in villages than cities, which supports the hypothesis of delaying vaccination, possibly related to more difficult access to vaccinations centers.

During all rounds, prior COVID-19 diagnosis was associated with increased willingness to vaccinate. One previous study reported similar results where individuals without prior COVID-19 diagnosis were more hesitant and resistant toward vaccination against COVID-19 (8). It is possible that those who have not been infected with COVID-19 might be less concerned about COVID-19, which then can lead to lower willingness to vaccinate (38). In addition, severe COVID-19 can be a significant motivator for vaccination against COVID-19, with adults experiencing mild symptoms being more hesitant to vaccination (39). This is also supported by our findings, that participants who were previously hospitalized with COVID-19 reported the highest willingness to receive the vaccine. The positive association between general hospitalization and willingness to vaccinate could indicate that people with health problems and therefore vulnerable to COVID-19, were more willing to get vaccinated to protect themselves against severe outcomes (40).

Exposure to COVID-19 was positively associated with willingness in round 1, whereas multiple contacts with infected individual(s) were negatively associated with willingness in rounds 2, and 4. In round 1 there was higher risk perception and fear around contracting COVID-19 which could have led to higher vaccine acceptance (41). In round 2, after the vaccination campaign, individuals may have felt safer and therefore were less fearful of getting infected. Likewise, after summer 2021, with the relaxation of restrictions and prevention measures, and with a perceived lower risk regarding Omicron infections, unvaccinated individuals may have felt less concerned, even after being in contact with infected individuals (40).

Additionally, participation in events, social groups, or trips was associated with decreased willingness to vaccinate through all four rounds. It has been reported that individuals who did not avoid contact with other people, did not keep minimum distance, or did not cover their mouth and nose in the public were more likely to be vaccine hesitant (7). People participating in events with other individuals may feel that COVID-19 is not a dangerous disease, perceive COVID-19 as low risk and therefore are less likely to get vaccinated (7, 8).

Moreover, the risk perception of a given health behavior or advice, in this case receiving a COVID-19 vaccination, can influence decision-making of individuals (42). People who think that they have higher risk of experiencing vaccine-related side effects may be more reluctant to receive a COVID-19 vaccine, even if they are worried about COVID-19 (42, 43). In combination with evolving dynamics and information about population groups at risk it could have contributed to higher vaccine hesitancy. A previous study in medical professionals in Poland indicated that low risk perception and lack of information about vaccines can make an individual resistant to persuasion about the importance of vaccination (44). The same study also pointed out the importance of accessibility and low cost in convincing people to get vaccinated. Even though accessibility was not a prevalent reason for reluctance in our study, it should be an important element of future vaccination campaigns. Vaccine knowledge and vaccine literacy can also impact willingness to vaccinate (42, 45). Those with higher level of vaccine literacy may be more willing to receive any vaccine than those with lower levels of vaccine literacy (42). It is possible that in our study those who remained hesitant toward vaccination may have lower overall vaccine literacy and knowledge about vaccine development and safety. Nevertheless, we did not assess perceptions toward vaccines in general in our study. Finally, mandatory vaccination, although successfully implemented for other viruses, may not have been beneficial for COVID-19 vaccination uptake (46). In the context of COVID-19, mandatory vaccinations were seen as limiting personal freedom and decision-making (46). In novel vaccines compulsory vaccinations may negatively influence vaccine uptake in the general population, where it has been shown that dialogue and detailed and targeted communication can be more beneficial in improving willingness to vaccinate (46). In Poland, COVID-19 vaccination certificates allowed more freedom to enter public spaces including restaurants and lifted the quarantine obligation. Even if not mandatory, these initial strategies could have also contributed to the decreasing trend in willingness to vaccinate that we observed in our study, especially once vaccine certificates were not needed.

### Strengths

This was the largest nationally representative, repeated cross-sectional study conducted in Poland to date. It collected data at four different time points after the National COVID-19 Immunization Program was introduced and spanning three different epidemic waves related to Alfa, Delta, and Omicron variants. Therefore, we were able to capture changes in attitudes toward COVID-19 vaccination as pandemic conditions were changing. Furthermore, we were able to assess changes in associations of several factors with willingness to vaccinate during this dynamic period. Stratification according to age and population distribution of each administrative region in Poland facilitated representativeness of our study sample. Apart from the addition of few questions at subsequent rounds, the same set of variables were collected in each round. This enabled us to compare findings between rounds and thus capture the impact of emerging variants, including Omicron.

### Limitations

Participants were not asked about the reasons for reluctance in round 1. Additionally, many participants did not provide a specific reason for their reluctance to vaccinate (“other reason”) and we were unable to further explore this response. However, we provided participants with several reasons in our questionnaire. Moreover, in rounds 2, 3, and 4 a subset of panel participants were included (independence of observations); however, bias is unlikely due to the dynamic changes in attitudes during the COVID-19 pandemic. Although we adjusted our analyses for several variables, we have not included all potential confounders. Therefore, residual confounding may still be present. Moreover, in our study, most participants were of Polish nationality, thus we were unable to examine willingness to vaccinate in other nationalities or ethnic minorities in Poland. Finally, it is possible that vaccinated and/or health-conscious participants were more willing to participate in the study and therefore our estimates may have been underestimated.

## Conclusions

We observed a decline in willingness to vaccinate among unvaccinated and vaccinated participants. Concerns around side effects, safety, overall effectiveness and against COVID-19 variants were the most prevalent reasons for reluctance to vaccinate. Several factors were associated with willingness to vaccinate, with COVID-19 diagnosis, and participation in social activities being consistently associated with willingness to vaccinate in all rounds. The Omicron wave significantly influenced attitudes toward vaccination. This study underscores the critical role of public health messaging based on ongoing monitoring of attitudes and the need for constant health communication about COVID-19 vaccines. Future research should also examine the influence of misinformation on vaccine attitudes over time and how it influences different groups of people, especially vulnerable and vaccine resistant groups.

## Data availability statement

The raw data supporting the conclusions of this article will be made available by the authors, without undue reservation.

## Ethics statement

The studies involving humans were approved by Bioethics Committee of the National Institute of Public Health NIH - National Research Institute (No. 5/2021 of 02/03/2021). The studies were conducted in accordance with the local legislation and institutional requirements. Written informed consent for participation was not required from the participants or the participants' legal guardians/next of kin because data collection for this study took place during a telephone interview. Before the interview participants were asked whether they consented to participate in the study. No identifiable data were collected in this study.

## Author contributions

EK developed the research proposal, conducted the statistical analysis, and wrote the manuscript. MR, MS, MC, and MS-T developed and contributed to the study protocol and reviewed the manuscript. All authors contributed to the article and approved the submitted version.
