# Supplementary material for: Willingness to vaccinate among adults, and factors associated with vaccine acceptance of COVID-19 vaccines in a nationwide study in Poland between March 2021 and April 2022
Source: Front Public Health. 2023 Dec 4;11:1235585. doi: 10.3389/fpubh.2023.1235585 (PMC10726053; doi:10.3389/fpubh.2023.1235585)
Supplement: Supplementary file 1 [file Table_1.docx]

Supplementary Material

**Supplemental Text 1 – Questionnaires**

**Round 1**

D1. What is your date of birth: __/__/__

D2. Gender

□ Male

□ Female

D3. Voivodeship (administrative region)

List of voivodeships

D4. District

*List of districts in the voivodeship [D3]*

D5. Municipalities

*List of municipalities in the district [D4]*

D6. Place of residence

*List of places in the municipality [D5]*

D7. What is your nationality?

□ Polish

□ Other, which?

D8. Number of members (including yourself) in the household?

*If D8>1 then ask D9. D8 must be > than D9*

D9. Number of children under the age of 18?

D10. What has been your work status since March 2020?

*Multiple answers available*

□ I work, I am employed

□ Self-employed

□ Student/studying/completing qualifications

□ Retirement pensioner

□ Disability pensioner

□ I am not working and I am not looking for a job (I perform domestic/family chores)

□ I am not working, I am unemployed

*If D10=1,2 then proceed to D11*

D11. What is your occupation?

*Excel list of 448 occupations*

*If D10-1,2 then proceed to D12*

D12. What was your main mode of work since March 2020?

□ Remote work all or most of the time

□ Hybrid work (stationary and remote on specific days)

□ Stationary or field work, in direct contact with people / clients

□ Stationary or field work, limited contact with people

□ I did not work due to downtime

P1. Since March 2020, have you been tested positive for SARS-CoV-2 infection in a PCR or antigen test?

□ Yes

□ No [go to question P3]

P2. Please enter the date of the last positive result (may be limited to month and year): __/___

P3. Did you have direct contact for at least 15 minutes with a person diagnosed with coronavirus during the period of their infectivity?

*These contacts do not include contacts with infected individuals while wearing a mask i.e., at least a FFP2 (N95) mask; we include contacts in a cloth mask or face shield*

□ Yes, once

□ Yes, repeatedly

□ I didn’t have any contact

P4. In the period from September 2020 to now, did you experience the following symptoms?

*Multiple answers available*

□ Fever

□ Cough

□ Dyspnea

□ Loss of smell/taste

□ Sore throat

□ Runny nose

□ Muscle pain / joint pain

□ Fatigue

□ Headache

□ Abdominal pain

□ Nausea/vomiting

□ Diarrhea

□ Rash

□ Conjunctivitis

□ Chills

□ Loss of appetite

□ Nosebleeds

□ Disturbances of consciousness

□ Other neurological symptoms

□ other, what?

No symptoms [go to question P6]

P5. Were you on sick leave due to these symptoms?

□ Yes

□ No

□ Not applicable

P6. Have you been hospitalized since the beginning of March 2020?

□ Yes

□ No [go to question P8]

P7. Were you hospitalized due to COVID-19 or a respiratory infection, e.g. pneumonia, bronchitis?

□ Yes

□ No

□ Not applicable

P8. In the period from March 2020, did you participate in an occasional event such as a wedding, communion, baptism, funeral?

□ Yes

□ No

P9. How many people, apart from your household members, would you include in the group of close family and friends with whom you met (e.g., for dinner, coffee, beer) at least once per month in the period starting from March 2020?

____

P10. In the period from March 2020, did you participate in regular sports, religious, artistic groups, or similar activities / meetings, not related to your work?

□ Yes

□ No

P11. In the period from March 2020, did you participate in an organized trip, e.g., a trip / camp, business trip, sports trip?

□ Yes

□ No

P12. Have you been vaccinated against COVID-19?

□ Yes, one dose

□ No, two doses

□ No

*If P12=1,2 then answer P12a*

P12a. Please enter the date of the last dose: __/____ *(month/year)*

*If P12=3, then answer P12b*

P12b. Do you plan to get vaccinated at the earliest possible date according to your age or occupation?

□ Yes

□ No

P13. Will you agree to participate in the second round of this study in approx. 2 months on the same terms?

□ Yes

□ No

In Rounds 2, 3, and 4 the same questionnaire from Round 1 was used, with some additional questions about vaccinations, vaccine acceptance and education (Rounds 3,4).

**Round 2**

**P12c.** Please indicate the reason for your reluctance to be vaccinated:

------------------------------------------------------------------------------------

**DP12c**. Please indicate the reason for your reluctance to vaccinate your child.

**Round 3- Additional questions**

D9a. What is your education?

□ Basic (primary school)

□ Gymnasium (secondary school)

□ Average

□ Bachelor’s degree, polytechnic

□ Master’s degree

P1a. How many times have you been found positive on a smear for infection?

----------------------------------------------------------------------------------------------

P2a_1_Pole. Date of first smear test

--/----- (MM/YYYY)

P2a_2_Pole. Date of second smear test

--/----- (MM/YYYY)

P2a_3_Pole. Date of third smear test

--/----- (MM/YYYY)

P2a_4_Pole. Date of fourth smear test

--/----- (MM/YYYY)

P2a_5_Pole. Date of fifth smear test

--/----- (MM/YYYY)

P12aa. Please provide the name of the last vaccine taken:

□ Astra Zeneca

□ Johnson & Johnson

□ Moderna

□ Pfizer

□ I don’t remember

[PA12c Question for participants of previous rounds who previously declared their reluctance to get vaccinated, but changed their mind in the current round - what influenced the change in decision?]

PA12c_1. My employer required the COVID-19 vaccination

□ Yes

□ No

PA12c_2. The vaccination certificate enabled me to carry out my plans, for example, to travel abroad.

□ Yes

□ No

PA12c_3. The severe course of COVID-19 in a person close to me convinced me that this disease can be life-threatening

□ Yes

□ No

PA12c_4. I became convinced of the effectiveness of the vaccine - fewer people got sick, etc.

□ Yes

□ No

PA12c_5. I have convinced myself of the safety of the vaccine - few people have had adverse effects after vaccination

□ Yes

□ No

PA12c_6. I was convinced by family members/acquaintances

□ Yes

□ No

PA12c_7. I was convinced by advertisements and campaigns encouraging vaccination

□ Yes

□ No

PA12c_8. There were new vaccination points and there were no more difficulties in signing up

□ Yes

□ No

PA12cc. Do you plan to get vaccinated with another dose of the vaccine in the next few months?

□ Yes

□ No

If no, why?

PB12c_1. I contracted COVID-19 despite being vaccinated

□ Yes

□ No

PB12c_2. I stopped believing in the effectiveness of the vaccine, too many vaccinated people got sick

□ Yes

□ No

PB12c_3. I felt bad after the previous dose of vaccine

□ Yes

□ No

PB12c_4. The vaccine is only effective for a short time and will not protect against variants.

□ Yes

□ No

PB12c_5. I have encountered difficulties enrolling at a vaccination center near my location

□ Yes

□ No

PB12c_6. I have difficulty getting to the vaccination center on my own/I am sick///unhealthy

□ Yes

□ No

PB12c_7. Doctor did not qualify me for vaccination for health reasons

□ Yes

□ No

PB12c_8. Other reason:

-----------------------------------------------------------------------------------------------------------

P12b. Do you plan to get vaccinated at the earliest possible time for you?

□ Yes

□ No

P12c_01. I am afraid of the side effects of the vaccine / I am afraid of an allergic reaction

□ Yes

□ No

P12c_02. Vaccine developed too fast, it can't be safe

□ Yes

□ No

P12c_03. The vaccine will only be effective for a short time and will not protect against the variants

□ Yes

□ No

P12c_04. As a rule I don't vaccinate, I don't trust pharmaceutical companies

□ Yes

□ No

P12c_05. I was sick with COVID-19

□ Yes

□ No

P12c_06. I have encountered difficulties enrolling at a vaccination center near my location

□ Yes

□ No

P12c_07. I have difficulty getting to the vaccination center on my own/ I am sick// unhealthy

□ Yes

□ No

P12c_08. I believe that COVID-19 is not a serious disease

□ Yes

□ No

P12c_09. I have a doctor's diagnosis of contraindication to vaccination

□ Yes

□ No

P12c_10. I believe that getting sick is more effective than getting vaccinated

□ Yes

□ No

P12c_11. Other reason:

-----------------------------------------------------------------------------------------------

**Round 4- Additional questions**

Q2A. Since March 2020 (all participants), have you tested positive on a COVID-19 antigen self-test (at-home test purchased in a store, online store, or pharmacy)?

□ Yes

□ No

Q11A. In the period from February 24, 2022, did you have contact with refugees from Ukraine?

□ Yes

□ No

**Supplemental Table 1:** Detailed list of confounders for each factor and regression model in Rounds 1-4.

|  | **Confounders** (X_round_=Included in the fully adjusted regression model) | | | | | | | | | | | | | | | | | | |
| --- | --- | --- | --- | --- | --- | --- | --- | --- | --- | --- | --- | --- | --- | --- | --- | --- | --- | --- | --- |
| **Factor** | Round | Age | Gender | Place of residence | Education | Pensioner | Household size | Work status | Remote work | COVID-19 diagnosis | Exposure to COVID-19 | Symptoms | Sick leave | General hospitalization | Hospitalization due to COVID-19 | Number of close contacts | Participation in events | Participation in social groups | Participation in organized trips |
| **Age** | 1 | - | X_1_ | X_1_ | - | - | X_1_ | X_1_ | X_1_ | X_1_ | - | X_1_ | - | - | X_1_ | X_1_ | X_1_ | X_1_ | X_1_ |
|  | 2 | - | - | X_2_ | - | - | - | - | - | X_2_ | X_2_ | X_2_ | - | - | - | - | X_2_ | X_2_ | X_2_ |
|  | 3 | - | X_3_ | X_3_ | - | - | - | - | - | X_3_ | - | - | X_3_ | - | - | X_3_ | X_3_ | X_3_ | X_3_ |
|  | 4 | - | X_4_ | - | X | - | - | X_4_ | - | - | X_4_ | X_4_ | - | X_4_ | - | X_4_ | X_4_ | - | - |
| **Gender** | 1 | X_1_ | - | X_1_ | - | - | X_1_ | X_1_ | X_1_ | X_1_ | - | - | - | - | X_1_ | X_1_ | - | X_1_ | X_1_ |
|  | 2 | X_2_ | - | X_2_ | - | - | - | - | - | X_2_ | X_2_ | X_2_ | - | - | - | - | X_2_ | X_2_ | X_2_ |
|  | 3 | X_3_ | - | X_3_ | - | X_3_ | - | X_3_ | - | X_3_ | - | X_3_ | - | - | - | X_3_ | X_3_ | X_3_ | X_3_ |
|  | 4 | - | - | - | X_4_ | - | - | X_4_ | - | X_4_ | X_4_ | X_4_ | - | X_4_ | - | X_4_ | X_4_ | - | X_4_ |
| **Size of place of residence** | 1 | X_1_ | - | - | - | - | X_1_ | X_1_ | X_1_ | X_1_ | - | X_1_ | - | - | - | X_1_ | - | X_1_ | X_1_ |
|  | 2 | X_2_ | - | - | - | - | - | - | - | X_2_ | X_2_ | X_2_ | - | - | - | X_2_ | X_2_ | X_2_ | X_2_ |
|  | 3 | X_3_ | X_3_ | - | - | - | - | X_3_ | - | X_3_ | - | - | X_3_ | - | - | X_3_ | X_3_ | X_3_ | X_3_ |
|  | 4 | - | X_4_ | - | X_4_ | - | X_4_ | X_4_ | - | X_4_ | X_4_ | - | X_4_ | X_4_ | - | X_4_ | X_4_ | - | - |
| **Work status** | 1 | X_1_ | - | X_1_ | - | - | X_1_ | - | X_1_ | X_1_ | - | - | - | - | - | X_1_ | - | X_1_ | - |
|  | 2 | X_2_ | - | X_2_ | - | - | - | - | - | X_2_ | X_2_ | X_2_ | - | - | - | X_2_ | X_2_ | X_2_ | X_2_ |
|  | 3 | X_3_ | X_3_ | X_3_ | - | X_3_ | - | - | - | X_3_ | - | - | X_3_ | - | - | X_3_ | X_3_ | X_3_ | X_3_ |
|  | 4 | - | X_4_ | - | X_4_ | - | X_4_ | - | - | X_4_ | X_4_ | - | X_4_ | X_4_ | - | X_4_ | X_4_ | - | - |
| **Remote work** | 1 | X_1_ | - | X_1_ | - | - | - | X_1_ | - | X_1_ | - | X_1_ | - | - | - | X_1_ | - | X_1_ | - |
|  | 2 | X_2_ | - | X_2_ | - | - | - | - | - | - | X_2_ | X_2_ | - | - | - | X_2_ | X_2_ | X_2_ | X_2_ |
|  | 3 | X_3_ | X_3_ | X_3_ | - | - | - | - | - | X_3_ | - | X_3_ | - | X_3_ | - | X_3_ | X_3_ | X_3_ | X_3_ |
|  |  | Round | Age | Gender | Place of residence | Education | Pensioner | Household size | Work status | Remote work | COVID-19 diagnosis | Exposure to COVID-19 | Symptoms | Sick leave | General hospitalization | Hospitalization due to COVID-19 | Number of close contacts | Participation in events | Participation in social groups |
|  | 4 | - | X_4_ | - | X_4_ | - | X_4_ | X_4_ | - | X_4_ | X_4_ | - | X_4_ | - | X_4_ | X_4_ | X_4_ | X_4_ | X_4_ |
| **COVID-19 diagnosis** | 1 | X_1_ | - | X_1_ |  | - | X_1_ | X_1_ | X_1_ | - | - | X_1_ | - | - | X_1_ | X_1_ | - | X_1_ | - |
|  | 2 | X_2_ | - | X_2_ |  | - | - | - | - | - | X_2_ | X_2_ | - | - | - | X_2_ | X_2_ | X_2_ | X_2_ |
|  | 3 | X_3_ | X_3_ | X_3_ |  | - | - | X_3_ | - | - | - | X_3_ | X_3_ | - | - | X_3_ | - | X_3_ | - |
|  | 4 | - | X_4_ | - | X_4_ | - | - | X_4_ | - | - | X_4_ | X_4_ | - | X_4_ | - | X_4_ | X_4_ | - | - |
| **Exposure to COVID-19** | 1 | X_1_ | - | X_1_ |  | - | X_1_ | X_1_ | X_1_ | X_1_ | - | X_1_ | - | - | X_1_ | X_1_ | - | X_1_ | - |
|  | 2 | X_2_ | - | X_2_ |  | - | - | - | - | X_2_ | - | X_2_ | - | - | - | X_2_ | X_2_ | X_2_ | X_2_ |
|  | 3 | X_3_ | X_3_ | X_3_ |  | - | - | X_3_ | - | - | - | X_3_ | - | - | - | X_3_ | X_3_ | X_3_ | X_3_ |
|  | 4 | - | X_4_ | - | X_4_ | - | X_4_ | - | - | X_4_ | - | X_4_ | - | - | X_4_ | X_4_ | X_4_ | X_4_ | X_4_ |
| **General hospitalization** | 1 | X_1_ | - | X_1_ |  | - | X_1_ | X_1_ | X_1_ | X_1_ | - | X_1_ | - | - | - | X_1_ | - | X_1_ | - |
|  | 2 | X_2_ | - | X_2_ |  | - | - | - | - | X_2_ | X_2_ | X_2_ | X_2_ | - | - | X_2_ | X_2_ | X_2_ | X_2_ |
|  | 3 | X_3_ | X_3_ | X_3_ |  | - | - | - | - | X_3_ | - | X_3_ | - | - | - | X_3_ | X_3_ | X_3_ | X_3_ |
|  | 4 | - | X_4_ | - | X_4_ | X_4_ | X_4_ | - | - | X_4_ | X_4_ | X_4_ | - | - | - | X_4_ | X_4_ | X_4_ | - |
|  |  |  |  |  |  | - |  |  |  |  |  |  |  |  |  |  |  |  |  |
| **Hospitalization due to COVID-19** | 1 | X_1_ | X_1_ | X_1_ |  | - | - | X_1_ | X_1_ | X_1_ | - | X_1_ | - | - | - | X_1_ | - | X_1_ | - |
|  | 2 | X_2_ | - | X_2_ |  | - | - | - | - | X_2_ | X_2_ | X_2_ | X_2_ | - | - | X_2_ | X_2_ | X_2_ | X_2_ |
|  | 3 | X_3_ | X_3_ | X_3_ |  | - | - | - | - | X_3_ | - | X_3_ | - | - | - | X_3_ | X_3_ | X_3_ | X_3_ |
|  | 4 | - | X_4_ | - | X_4_ | - | X_4_ | X_4_ | - | X_4_ | X_4_ | X_4_ | - | - | - | X_4_ | - | X_4_ | X_4_ |
| **Participation in events** | 1 | X_1_ | - | X_1_ |  | - | X_1_ | X_1_ | X_1_ | X_1_ | - | X_1_ | - | - | - | X_1_ | - | X_1_ | X_1_ |
|  | Round | Age | Gender | Place of residence | Education | Pensioner | Household size | Work status | Remote work | COVID-19 diagnosis | Exposure to COVID-19 | Symptoms | Sick leave | General hospitalization | Hospitalization due to COVID-19 | Number of close contacts | Participation in events | Participation in social groups | Participation in organized trips |
|  | 2 | X_2_ | - | X_2_ |  | - | - | - | - | X_2_ | X_2_ | X_2_ | - | - | - | X_2_ | - | X_2_ | X_2_ |
|  | 3 | X_3_ | X_3_ | X_3_ |  | - | - | - | - | X_3_ | - | - | - | X_3_ | - | X_3_ | - | X_3_ | X_3_ |
|  | 4 | - | X_4_ | - | X_4_ | - | - | - | - | X_4_ | X_4_ | X_4_ | - | - | X_4_ | X_4_ | - | X_4_ | X_4_ |
| **Participation in social groups** | 1 | X_1_ | X_1_ | X_1_ |  | - | X_1_ | X_1_ | X_1_ | X_1_ | X_1_ | X_1_ | - | - | - | X_1_ | - | - | X_1_ |
|  | 2 | X_2_ | - | X_2_ |  | - | - | - | - | X_2_ | X_2_ | X_2_ | - | - | - | X_2_ | X_2_ | - | X_2_ |
|  | 3 | - | X_3_ | - |  | X_3_ | - | X_3_ | - | X_3_ | - | - | - | X_3_ | - | X_3_ | X_3_ | - | X_3_ |
|  | 4 | - | X_4_ | - | X_4_ | - | - | X_4_ | - | X_4_ | X_4_ | X_4_ | - | - | - | X_4_ | X_4_ | - | X_4_ |
| **Participation in organized trips** | 1 | X_1_ | X_1_ | X_1_ |  | - | X_1_ | X_1_ | X_1_ | X_1_ | X_1_ | X_1_ | - | - | - | X_1_ | - | X_1_ | - |
|  | 2 | - | - | X_2_ |  | X_2_ | - | - | - | X_2_ | X_2_ | X_2_ | - | - | - | X_2_ | X_2_ | X_2_ | - |
|  | 3 | X_3_ | X_3_ | X_3_ |  | - | - | X_3_ | - | X_3_ | X_3_ | - | - | - | - | X_3_ | X_3_ | X_3_ | - |
|  | 4 | - | X_4_ | - | X_4_ | - | - | X_4_ | - | X_4_ | X_4_ | - | - | - | - | X_4_ | X_4_ | X_4_ | - |

**Supplementary Table 2.** Crude and adjusted odds ratios and 95% CI of associations of several factors with willingness to vaccinate among unvaccinated participants in rounds 1-4.

|  | **Willingness to get vaccinated** | | | | | | | | |
| --- | --- | --- | --- | --- | --- | --- | --- | --- | --- |
|  | Round 1 (n=15,885) | | Round 2 (n=4,006) | | Round 3 (n=3,044) | | Round 4 (n=4,023) | | |
|  | OR | (95% CI) | OR | (95% CI) | OR | (95% CI) | OR | | (95% CI) |
| **Age in years** |  |  |  |  |  |  |  | |  |
|  | **Crude model** | | | | | | | | |
|  |  | | | | | | | | |
| 20-39 | *ref* | *ref* | *ref* | *ref* | *ref* | *ref* | | *ref* | *ref* |
| 40-59 | 1.69 | (1.53, 1.87) | 1.21 | (1.00, 1.45) | 1.24 | (1.00, 1.54) | | 1.06 | (0.81, 1.39) |
| 60-69 | 2.01 | (1.78, 2.28) | 1.40 | (1.10, 1.78) | 1.67 | (1.26, 2.20) | | 1.00 | (0.71, 1.42) |
| ≥70 | 1.78 | (1.48, 2.15) | 1.40 | (1.02, 1.93) | 1.17 | (0.81, 1.69) | | 1.10 | (0.69, 1.77) |
|  | **Fully adjusted model** | | | | | | | | |
| 20-39 | *ref* | *ref* | *ref* | *ref* | *ref* | *ref* | *ref* | | *ref* |
| 40-59 | 1.79 | (1.62, 1.98) | 1.16 | (0.95, 1.40) | 1.17 | (0.93, 1.46) | 0.93 | | (0.71, 1.22) |
| 60-69 | 1.98 | (1.69, 2.31) | 1.26 | (0.99, 1.61) | 1.47 | (1.10, 1.95) | 0.77 | | (0.50, 1.18) |
| ≥70 | 1.60 | (1.28, 1.99) | 1.23 | (0.88, 1.71) | 0.91 | (0.61, 1.36) | 0.71 | | (0.40, 1.24) |
|  |  |  |  |  |  |  |  | |  |
| **Gender** | **Crude model** | | | | | | | | |
| Man | *ref* | *ref* | *ref* | *ref* | *ref* | *ref* | *ref* | | *ref* |
| Woman | 0.96 | (0.90, 1.04) | 1.15 | (1.01, 1.31) | 1.39 | (1.19, 1.62) | 1.29 | | (1.06, 1.55) |
|  | **Fully adjusted model** | | | | | | | | |
| Man | *ref* | *ref* | *ref* | *ref* | *ref* | *ref* | *ref* | | *ref* |
| Woman | 0.89 | (0.82, 0.96) | 0.99 | (0.86, 1.13) | 1.25 | (1.06, 1.48) | 1.11 | | (0.91, 1.36) |
|  |  |  |  |  |  |  |  | |  |
| **Place of residence** | **Crude model** | | | | | | | | |
| Village | *ref* | *ref* | *ref* | *ref* | *ref* | *ref* | *ref* | | *ref* |
| City up to 50,000 inhabitants | 1.08 | (0.96, 1.21) | 0.83 | (0.67, 1.02) | 0.73 | (0.57, 0.93) | 0.94 | | (0.70, 1.27) |
| City 50,000-100,000 inhabitants | 1.10 | (0.92, 1.30) | 0.72 | (0.53, 0.98) | 0.73 | (0.51, 1.03) | 0.79 | | (0.48, 1.30) |
| City >100,000 inhabitants | 1.36 | (1.23, 1.51) | 0.65 | (0.54, 0.80) | 0.82 | (0.66, 1.03) | 0.83 | | (0.62, 1.11) |
|  | **Fully adjusted model** | | | | | | | | |
| Village | *ref* | *ref* | *ref* | *ref* | *ref* | *ref* | *ref* | | *ref* |
| City up to 50,000 inhabitants | 1.00 | (0.89, 1.13) | 0.78 | (0.63, 0.96) | 0.70 | (0.55, 0.89) | 0.88 | | (0.64, 1.21) |
| City 50,000-100,000 inhabitants | 1.03 | (0.85, 1.25) | 0.71 | (0.52, 0.99) | 0.72 | (0.49, 1.04) | 0.73 | | (0.45, 1.18) |
| City >100,000 inhabitants | 1.27 | (1.12, 1.45) | 0.65 | (0.53, 0.79) | 0.82 | (0.64, 1.04) | 0.82 | | (0.60, 1.12) |
|  |  |  |  |  |  |  |  | |  |
|  | Round 1 (n=15,885) | | Round 2 (n=4,006) | | Round 3 (n=3,044) | | Round 4 (n=4,023) | | |
|  | OR | (95% CI) | OR | (95% CI) | OR | (95% CI) | OR | | (95% CI) |
| **Work status** | **Crude model** | | | | | | | | |
| Employed | *ref* | *ref* | *ref* | *ref* | *ref* | *ref* | *ref* | | *ref* |
| Unemployed | 0.74 | (0.65, 0.84) | 1.28 | (1.02, 1.60) | 1.30 | (1.06, 1.82) | 1.79 | | (1.38, 2.32) |
|  | **Fully adjusted model** | | | | | | | | |
| Employed | *ref* | *ref* | *ref* | *ref* | *ref* | *ref* | *ref* | | *ref* |
| Unemployed | 0.90 | (0.78, 1.03) | 1.09 | (1.05, 1.13) | 1.21 | (0.90, 1.63) | 1.46 | | (1.05, 2.01) |
|  |  |  |  |  |  |  |  | |  |
|  |  |  |  |  |  |  |  | |  |
| **Remote work** | **Crude model** | | | | | | | | |
| No | *ref* | *ref* | *ref* | *ref* | *ref* | *ref* | *ref* | | *ref* |
| Yes | 1.56 | (1.43, 1.70) | 0.95 | (0.79, 1.13) | 0.90 | (0.71, 1.14) | 0.84 | | (0.64, 1.10) |
|  | **Fully adjusted model** | | | | | | | | |
| No | *ref* | *ref* | *ref* | *ref* | *ref* | *ref* | *ref* | | *ref* |
| Yes | 1.79 | (1.63, 1.98) | 1.16 | (0.96, 1.41) | 1.01 | (0.79, 1.31) | 1.16 | | (0.85, 1.57) |
|  |  |  |  |  |  |  |  | |  |
| **COVID-19 diagnosis** | **Crude model** | | | | | | | | |
| No | *ref* | *ref* | *ref* | *ref* | *ref* | *ref* | *ref* | | *ref* |
| Yes | 1.50 | (1.36, 1.67) | 1.73 | (1.44, 2.09) | 1.44 | (1.20, 1.72) | 1.37 | | (1.12, 1.67) |
|  | **Fully adjusted model** | | | | | | | | |
| No | *ref* | *ref* | *ref* | *ref* | *ref* | *ref* | *ref* | | *ref* |
| Yes | 1.35 | (1.21, 1.50) | 1.55 | (1.26, 1.92) | 1.38 | (1.13, 1.70) | 1.28 | | (1.02, 1.59) |
|  |  |  |  |  |  |  |  | |  |
| **Contact with infected individual** | **Crude model** | | | | | | | | |
| No contact | *ref* | *ref* | *ref* | *ref* | *ref* | *ref* | *ref* | | *ref* |
| Once | 1.16 | (1.02, 1.32) | 1.21 | (0.92, 1.59) | 0.91 | (0.67, 1.24) | 0.93 | | (0.67, 1.30) |
| Multiple | 0.99 | (0.90, 1.10) | 0.62 | (0.50, 0.76) | 0.73 | (0.56, 0.97) | 0.52 | | (0.39, 0.71) |
|  | **Fully adjusted model** | | | | | | | | |
| No contact | *ref* | *ref* | *ref* | *ref* | *ref* | *ref* | *ref* | | *ref* |
| Once | 1.17 | (1.02, 1.34) | 1.18 | (0.88, 1.59) | 0.96 | (0.69, 1.33) | 1.00 | | (0.71, 1.41) |
| Multiple | 1.07 | (0.95, 1.20) | 0.67 | (0.53, 0.83) | 0.92 | (0.69, 1.24) | 0.62 | | (0.46, 0.85) |
|  |  |  |  |  |  |  |  | |  |
|  |  |  |  |  |  |  |  | |  |
| **General hospitalization** | **Crude model** | | | | | | | | |
| No | *ref* | *ref* | *ref* | *ref* | *ref* | *ref* | *ref* | | *ref* |
| Yes | 1.35 | (1.19, 1.54) | 1.20 | (0.98, 1.47) | 1.43 | (1.15, 1.79) | 1.57 | | (1.22, 2.06) |
|  | **Fully adjusted model** | | | | | | | | |
| No | *ref* | *ref* | *ref* | *ref* | *ref* | *ref* | *ref* | | *ref* |
| Yes | 1.26 | (1.10, 1.44) | 1.06 | (0.81, 1.39) | 1.31 | (1.04, 1.65) | 1.46 | | (1.12, 1.91) |
|  |  |  |  |  |  |  |  | |  |
|  | Round 1 (n=15,885) | | Round 2 (n=4,006) | | Round 3 (n=3,044) | | Round 4 (n=4,023) | | |
|  | OR | (95% CI) | OR | (95% CI) | OR | (95% CI) | OR | | (95% CI) |
| **Hospitalization due to COVID-19** | **Crude model** | | | | | | | | |
| No | *ref* | *ref* | *ref* | *ref* | *ref* | *ref* | *ref* | | *ref* |
| Yes | 2.72 | (1.76, 4.20) | 2.16 | (1.22, 3.84) | 2.09 | (1.03, 4.25) | 3.13 | | (1.94, 5.04) |
|  | **Fully adjusted model** | | | | | | | | |
| No | *ref* | *ref* | *ref* | *ref* | *ref* | *ref* | *ref* | | *ref* |
| Yes | 1.77 | (1.13, 2.76) | 1.14 | (0.62, 2.10) | 1.50 | (0.73, 3.12) | 2.36 | | (1.40, 3,97) |
|  |  |  |  |  |  |  |  | |  |
| **Participation in events/parties** | **Crude model** | | | | | | | | |
| No | *ref* | *ref* | *ref* | *ref* | *ref* | *ref* | *ref* | | *ref* |
| Yes | 0.78 | (0.73, 0.84) | 0.65 | (0.57, 0.73) | 0.68 | (0.58, 0.79) | 0.54 | | (0.44, 0.67) |
|  | **Fully adjusted model** | | | | | | | | |
| No | *ref* | *ref* | *ref* | *ref* | *ref* | *ref* | *ref* | | *ref* |
| Yes | 0.94 | (0.86, 1.01) | 0.79 | (0.68, 0.91) | 0.85 | (0.72, 1.00) | 0.73 | | (0.59, 0.91) |
|  |  |  |  |  |  |  |  | |  |
| **Participation in social groups** | **Crude model** | | | | | | | | |
| No | *ref* | *ref* | *ref* | *ref* | *ref* | *ref* | *ref* | | *ref* |
| Yes | 0.61 | (0.56, 0.66) | 0.57 | (0.49, 0.66) | 0.61 | (0.51, 0.72) | 0.59 | | (0.48, 0.72) |
|  | **Fully adjusted model** | | | | | | | | |
| No | *ref* | *ref* | *ref* | *ref* | *ref* | *ref* | *ref* | | *ref* |
| Yes | 0.63 | (0.58, 0.68) | 0.70 | (0.60, 0.81) | 0.74 | (0.62, 0.88) | 0.81 | | (0.66, 1.01) |
|  |  |  |  |  |  |  |  | |  |
| **Participation in organized trips** | **Crude model** | | | | | | | | |
| No | *ref* | *ref* | *ref* | *ref* | *ref* | *ref* | *ref* | | *ref* |
| Yes | 0.77 | (0.70, 0.85) | 0.56 | (0.48, 0.66) | 0.62 | (0.51, 0.75) | 0.53 | | (0.41, 0.70) |
|  | **Fully adjusted model** | | | | | | | | |
| No | *ref* | *ref* | *ref* | *ref* | *ref* | *ref* | *ref* | | *ref* |
| Yes | 0.87 | (0.78, 0.96) | 0.75 | (0.62, 0.90) | 0.80 | (0.65, 0.98) | 0.78 | | (0.59, 1.04) |

**
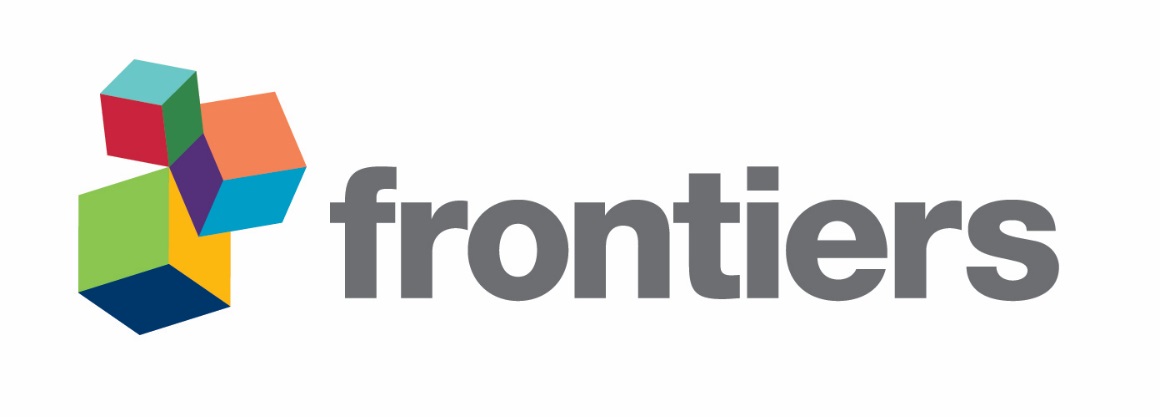
**
